# Evaluation of a sticky trap (AedesTraP), made from disposable plastic bottles, as a monitoring tool for *Aedes aegypti* populations

**DOI:** 10.1186/1756-3305-5-195

**Published:** 2012-09-07

**Authors:** Eloína Maria Mendonça de Santos, Maria Alice Varjal de Melo-Santos, Claudia Maria Fontes de Oliveira, Juliana Cavalcanti Correia, Cleide Maria Ribeiro de Albuquerque

**Affiliations:** 1 Laboratory of Terrestrial Invertebrates, Department of Zoology, Center of Biological Sciences, Universidade Federal de Pernambuco. Av. Moraes Rego s/n, Cidade Universitária, Recife, PE, CEP: 50670-420, Brazil; 2 Department of Entomology, Centro de Pesquisas Aggeu Magalhães, Fundação Oswaldo Cruz. Av. Moraes Rego s/n, Cidade Universitária, Recife, PE, CEP: 50670-420, Brazil

**Keywords:** *Aedes* female, Sticky trap, Locality, Ovitrap, Surveillance

## Abstract

**Background:**

Dengue virus, which is transmitted by *Aedes aegypti* mosquitoes is the most important emerging viral disease, infecting more than 50 million people annually. Currently used sticky traps are useful tools for monitoring and control of *A. aegypti*, despite differences in efficiency, labor requirements and cost. In the present work, a field assay was carried out to evaluate the performance of a sticky trap (AedesTrap), produced using disposable material, in capturing gravid *Aedes* spp. females. Additionally, conditions necessary for the improved performance of the device, such as number of traps per site and location (indoors or outdoors) were evaluated.

**Methods:**

During a one year period, traps were placed in a dengue endemic area in 28 day cycles. The trap, named AedesTrap, consisted of a disposable plastic soda bottle coated inside with colophony resin, which served as a sticky substrate. Disposable bottles were donated by restaurants, and traps were made by laboratory staff, reducing the cost of the sticky trap (less than U$3). Mosquito capture in indoor and outdoor areas was compared by placing the traps in laundry room, kitchen or bedroom (indoors) and front or back yard (outdoors). The relationship between the number of AedesTraps and quantity of captured mosquitoes was investigated by utilizing one or three traps/site.

**Results:**

During a 28 day cycle, a single AedesTrap was capable of capturing up to 15 *A. aegypti* in a house, with a mean capture of 0.5 to 2.63 females per premise. The AedesTrap collected three times more outdoors versus indoors. Similarly, the capability of detecting *Aedes* spp. infestation, and of capturing females, was three times higher when using three AedesTraps per house, compared with one trap per house.

**Conclusions:**

AedesTrap was shown to be capable of capturing *A. aegypti* and other culicidae, providing information on the adult mosquito population, and allowing the identification of areas critically infested by mosquitoes. Low requirements for skilled labor together with easy maintenance and low cost are additional advantages of using this sticky trap.

## Background

As the most effective vector of important arboviruses such as yellow fever and dengue, *Aedes aegypti* (Linnaeus, 1762) represents a major threat to health in tropical regions. Despite the efforts of scientists and health service professionals, current methods for the surveillance and control of this mosquito species have been inadequate, requiring the development of new approaches for the control of the insect [1].

Conventional methods of monitoring and controlling *A. aegypti* differ in efficiency and labor requirements. Collection techniques such as backpack aspirators [2,3] and the BG-Sentinel [4], provide reliable samples, however, both require a power source and are highly labor intensive, rendering daily mosquito collection in dengue endemic areas not cost-effective [5,6]. Furthermore, several aspects, such as operator competence, site of collection (e.g. indoors vs. outdoors), site size, the presence of furniture, and the duration of sampling may influence manual collection results [5,6]. Some mosquito traps have been developed specifically to capture gravid *A. aegypti*. Sticky ovitraps [5,7] and the Adultrap [8,9] are examples of traps for *A. aegypti* females. An alternative strategy using adhesive material to capture the female during oviposition are different models of single sticky ovitraps [5,7,10] and double sticky traps [7,11]. These are based on ovitraps, which are originally made from black jars filled with water and provided with a hardboard paddle on which females laid their eggs [12]. Ovitraps are inexpensive and simple to assemble and operate. They are widely used to obtain information derived from number of eggs laid, and to assess the spatial/temporal distribution of mosquitoes [5,13].

Sticky traps are used to easily identify the species of mosquito and allow a direct count of the number of adults that have visited the trap. These traps have also been used to monitor mosquito population dynamics [5] and to investigate the ecological parameters of *A. aegypti* and *Aedes albopictus* in relation to eco-climatic factors [14,15]. In addition, dengue surveillance [16,17], dispersal of dengue vectors [3,16,18,19] and evaluation of the effectiveness of vector monitoring strategies [14] have been performed using sticky traps. The effective performance of sticky traps is a stimulus to the development of low-cost models to be used more widely by health services. The present study reports the results of field trials designed to evaluate the performance of sticky traps (AedesTrap), made from disposable bottles, in capturing *Aedes* spp. Conditions under which the AedesTrap use could be improved are also described.

## Methods

### Study area

The study was carried out in Engenho do Meio, an urban area with an area of 0.89 km^2^ and a population density of 11,865 habitants/Km^2^, situated in the city of Recife (22^o^ 03’ 20.7” S 34° 56' 43.6"W), in the north east of Brazil. The study area included mainly residential properties, usually with a back or front yard, covered with vegetation. Although control activities based on larval surveys, larvicides application, environmental management, education and mobilization have been performed in the area since 1996, by the National Program of Dengue Control (PNCD), entomological surveillance data shows that it has been continuously infested by *A. aegypti*[19]*.* An intermittent water supply, deprived sanitation conditions, high temperatures (ranging from 22°C to 32°C) and relative air humidity (70% – 90%) throughout the year, provide excellent conditions for mosquitoes to breed, which explains the continued presence of *A. aegypti* in the study area [19]. During the present study, weather variables were obtained from the National Institute of Meteorology (INMET), situated approximately 3.5 km from the study area.

### Trap design

**Figure 1:**
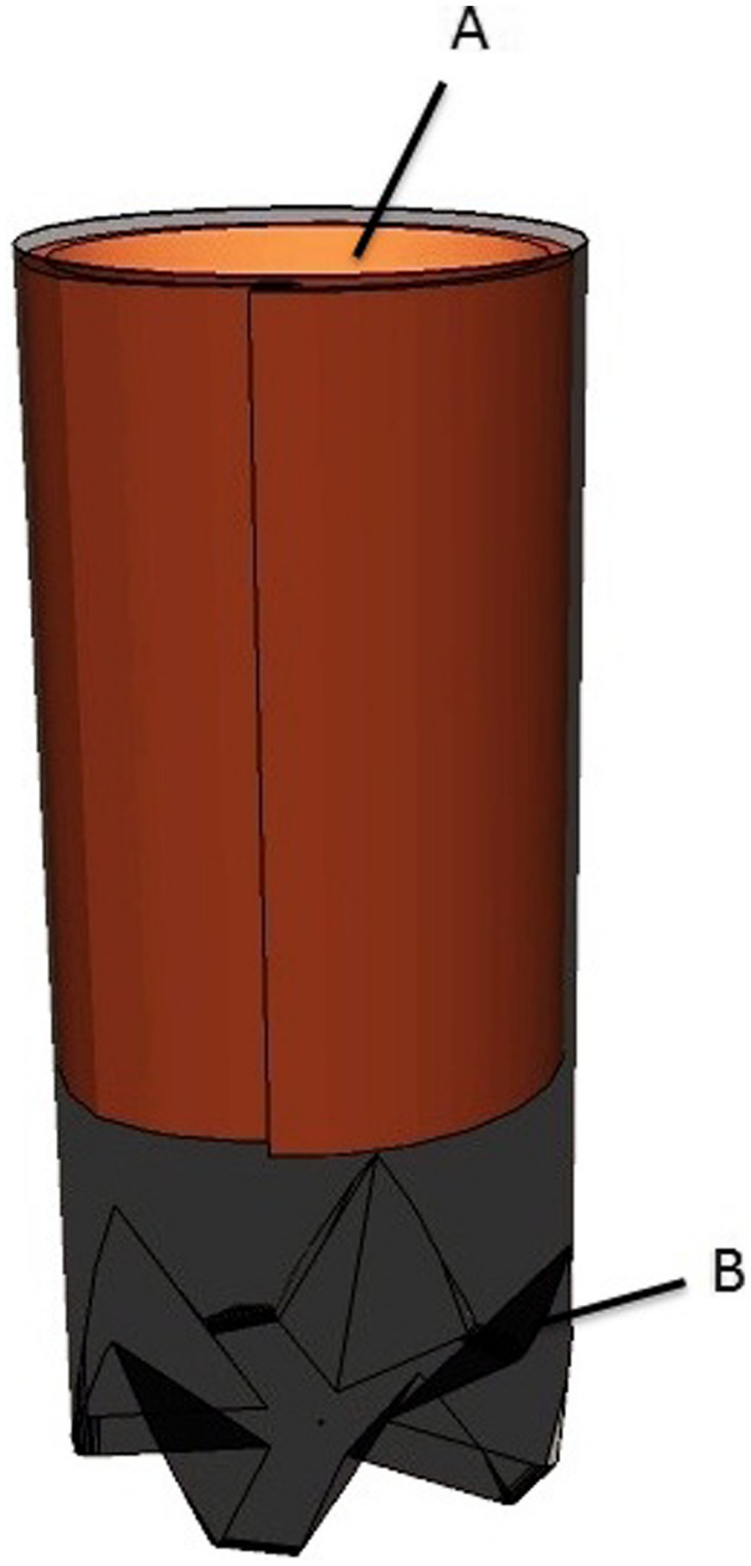
**Diagram of the AedesTrap. (A)** Stick substrate; **(B)** Plastic soda bottle.

The design of the trap for the collection of adults consisted of a disposable plastic soda bottle (2 L), 20 cm in length and 10 cm in width, painted black on the outside. Rubber material, 20 cm in length and coated on one side with colophony resin was placed inside the bottle, and served as a sticky substrate. This trap model was named the AedesTrap (Figure 1). Traps were labeled showing date and trap number.

A second group of traps used in this study were ovitraps, modified from a model utilized by Recife City Council. This trap was made from a disposable plastic soda bottle (2 L), painted black and containing two wooden paddles (5x12cm) fixed vertically with metal clips, serving as an oviposition substratum.

Both trap types were filled with 1.5 L of tap water with 2 mg of *Bacillus thuringiensis israelensis* granulated formulation (Bti), used as a larvicide to prevent the trap from becoming a breeding site. During the experiments, water, paddles and Bti were replaced every 28 days.

### Entomological survey

**Figure 2:**
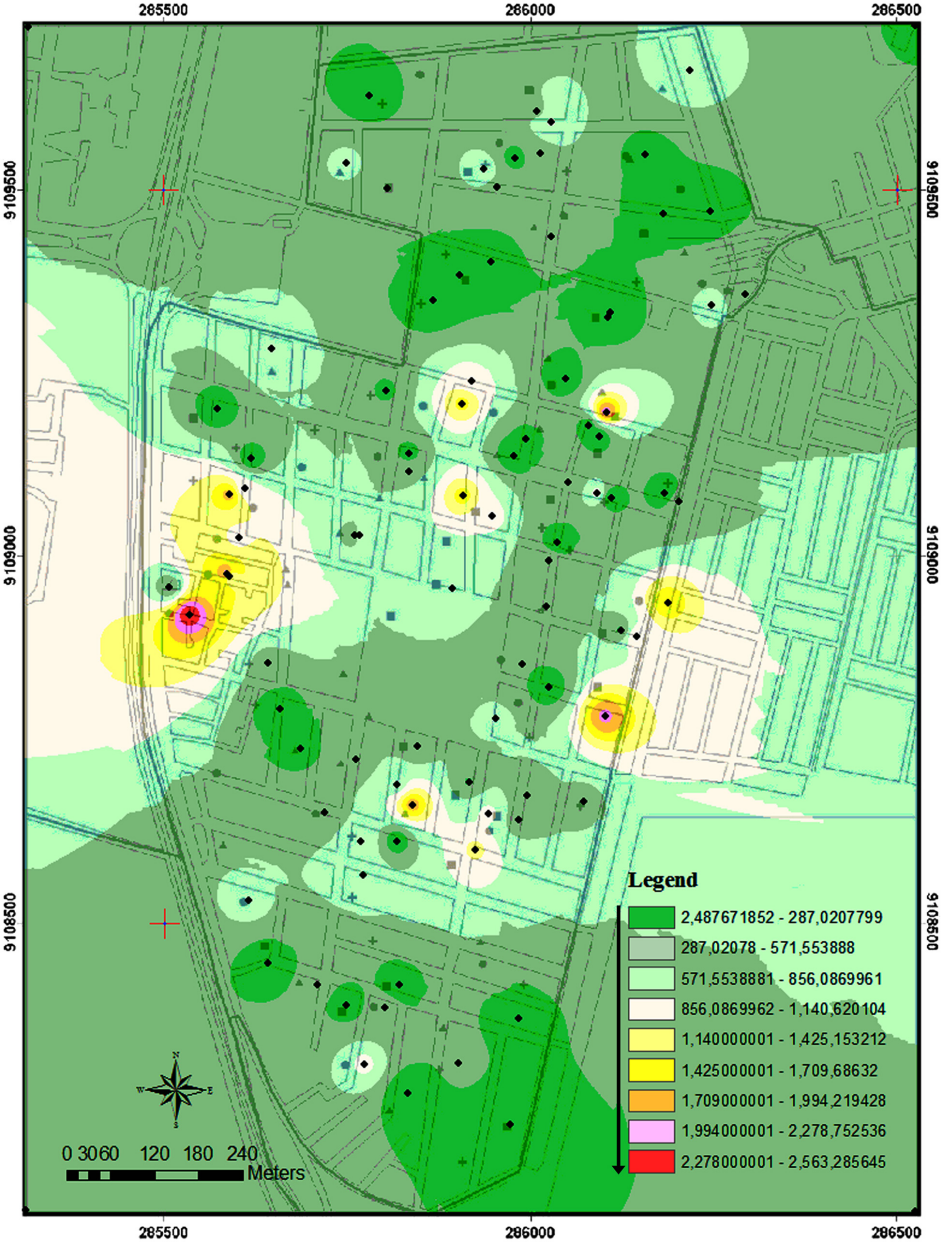
**Kernel map for spatial distribution of *Aedes aegypti *infestation in an endemic area of dengue.** Data were based on egg densities (100 ovitraps sampled in two cycles of 28 days) in the neighborhood of Engenho do Meio, Recife, Pernambuco, between September 2008 and November 2008. Arrow indicates egg density gradient in the area.

The infestation level of *Aedes* spp in the study area was preliminarily evaluated based on the egg densities from 100 ovitraps (one per premise) distributed in the area, according to Regis et al. [19]. The inspection procedures of the entomological survey were verbally explained to residents. A statement of informed consent was obtained from residents and/or householders who allowed the installation of traps on their properties. The traps were installed in partially shaded outdoor areas (service area, open garage) georeferenced (Garmin model TREX VISTA HCX), and monitored from September 2008 to November 2008. Adult *Aedes* spp. frequency was estimated from a sample of 30% of eggs collected in the ovitraps. Mosquito identification was performed, using the taxonomy key for adults described in Consoli and Lourenço-de-Oliveira [20]. The high dispersion of *Aedes* eggs (95% of positive traps) and heterogeneous egg distribution ranging from 1 to 3,439/eggs/ovitrap (mean = 280.41 ± 150.61 eggs/ovitrap) in the study area (Figure 2) allowed the random use of the area for experiments. Out of 758 identified mosquitoes obtained from the sampled eggs, 94.4% were *A. aegypti* and 5.6% were *Aedes albopictus* (Skuse, 1894).

### AedesTrap: performance in the field

#### Detecting and capturing *A. aegypti* females

57 premises were assessed (>100 eggs in the previous infestation assay) from November 2008 to March 2009. Each site, received an AedesTrap that remained in the field for a 16 week period. The installation of the AedesTrap was carried out in sets of 12–13 premises per week, over a period of 28 days. Thus, four groups [1-4] of sites were formed, differing from each other only by trap installation period (first, second, third or fourth week). The first set of AedesTraps was installed on November 13 and the last on March 26. Each set of traps was monitored every 28 days, with four observation cycles being performed.

In order to confirm the infestation of a site an ovitrap was installed at each premise, serving as a sentinel trap (OVT-S), as described in Regis et al. [19]. In all the experiments, traps were installed in shaded locations protected from wind and rain, at 1 m above the floor and 2 m apart from each other.

During the study period, precipitation was registered per group/cycle, ranging from 6.4 mm to 45.1 mm.

#### Mosquito capture in indoor and outdoor areas

The effect of AedesTrap installation (indoor or outdoor) on the capture of adults was evaluated at 28 premises, in which initial egg densities varied from 180 to 2,600 eggs/ovitrap/cycle. One AedesTrap was installed indoors (laundry room, kitchen or bedroom) and one AedesTrap outdoors front or back yard protected by roof or tree at each premise. One OVT-S was installed in the outdoor area of each premise as an additional tool to confirm the presence of *Aedes* spp. The experiment was performed from April to July 2009, with traps being monitored in four 28 day cycles.

#### Mosquito capture with different numbers of AedesTraps

The relationship between the number of AedesTraps and number of captured mosquitoes was investigated by utilizing one or three traps/premise placed outdoors at 24 and 22 premises, respectively. The experiments were carried out in four cycles between April 2009 and August 2009. At time of installation, the infestation level at these sites, estimated by egg density/ovitrap/cycle, varied from 70 to 1,772 eggs at sites with one AedesTrap, and from 60 to 1,400 eggs at sites with three traps. One OVT-S was also installed in the outdoor area of each premise.

To verify whether the presence and the number of AedesTraps would influence the performance of the ovitrap, egg density from the ovitraps used in the above experiments was compared with 20 ovitraps installed at sites without an AedesTrap.

### Data analysis

The proportion of positive traps served as the parameter to calculate the positivity index, reflecting the sensitivity of the trap. Traps were considered positive if they had at least one *Aedes* egg in the case of ovitraps and at least one *A. aegypti* adult in the case of AedesTraps. Trap efficiency was measured using the mean number ± standard error (SE) of collected eggs or adults, calculated by the total number of individuals and divided by the total number of installed traps per cycle. Data normality was determined using the Shapiro-Wilk test and homogeneity of variance was tested using the Levene test. Normality and homogeneity for eggs distribution in group 4 were achieved using natural logarithm of values plus average. The comparative analysis of collected adults and eggs was performed using Analysis of Variance (ANOVA) and Tukey test a posteriori. The relationship between egg and female densities were analyzed using the Spearman correlation coefficient. All analyses were performed with the STATISTICA 7.1 software, at a 5% significant level.

## Results

### AedesTrap performance

#### Detecting and capturing *A. aegypti*

The positivity index of AedesTraps varied from 5.9% to 57.1% with the average number of captured females of 0.54 ± 0.07 females/trap/cycle. There was no significant variation in the number of *A. aegypti* females captured among cycles and groups during this experiment. During the experiment (November 2008 to March 2009) cumulative precipitation for each cycle varied from 6.4 mm to 452.1 mm.

**Figure 3:**
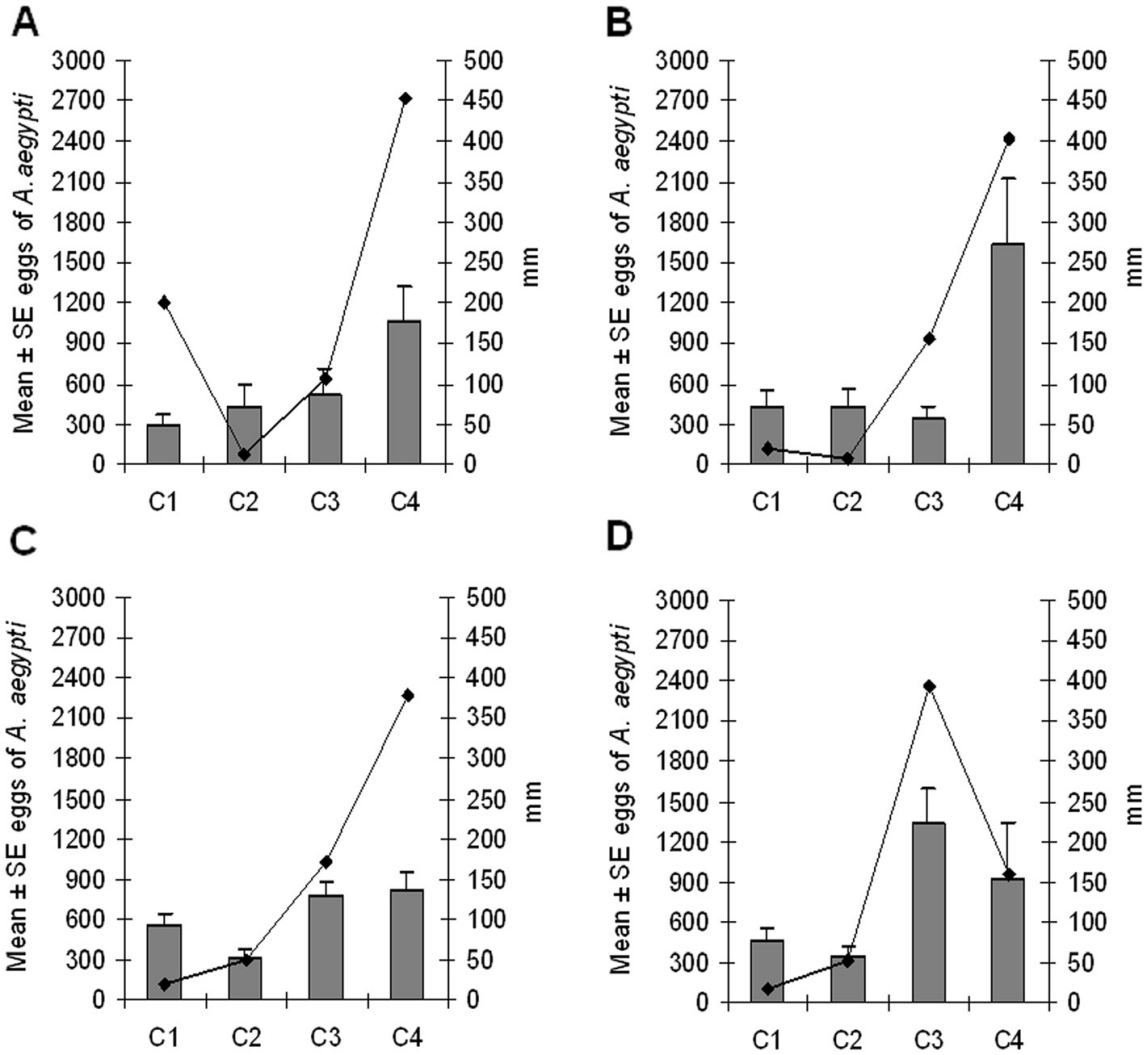
**Precipitation and *A. aegypti *eggs densities in the neighborhood of Engenho do Meio, Recife, Pernambuco.** Data are from November 2008 to March 2009. **A**, **B**, **C** and **D** represent groups of 12 or 13 ovitraps differing from each other only by trap installation period (first, second, third or fourth week). Each group was assessed in four cycles of 28 days. Line represents rainfall (mm) in the period. Bars are standard errors.

In the same period, 71% to 100% of ovitraps were positive. No significant difference was found in the mean number of eggs collected among the four groups. However in Group 3 significantly fewer eggs were registered in cycle 2 compared to cycles 3 and 4 (F = 5.4661; GL = 3,60; p < 0.005). Similar results were obtained in Group 4, were register significantly great number of eggs in cycle 3 compared to cycles 1 and 2 (F = 6,50; GL = 3,39;p < 0.005) (Figure 3).

**Figure 4:**
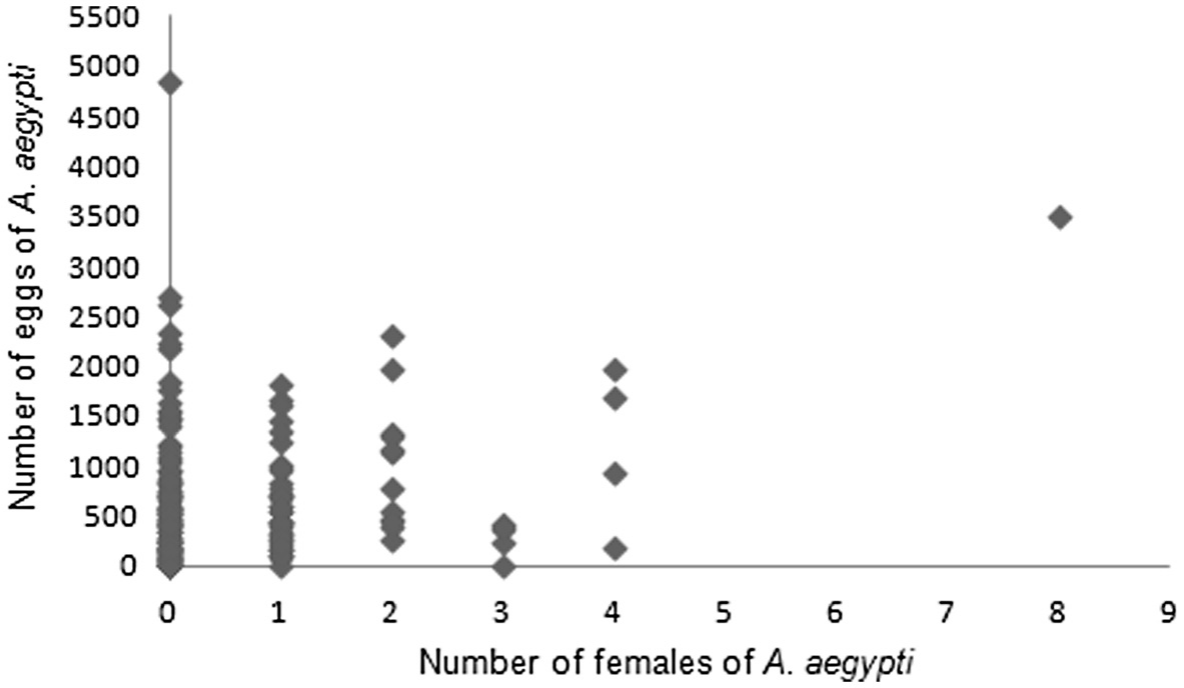
**Correlation between eggs density and female capture.** Spearman correlation between * A. aegypti * eggs and *A. aegypti* females collected in periods with distinct rainfall, during the period November 2008 to March 2009 in the neighborhood of Engenho do Meio neighborhood, Recife, Pernambuco. (N = 196, r = 0.2519, p < 0.05).

In general, egg density increased in correlation with increasing rainfall (Figure 2). The total number of eggs collected was 1,627.9 ± 495.8/trap (Group 2; cycle 4) and 1,340.25 ± 249.34/trap (Group 4; cycle 3) when the rainfall of 403.3 mm/cycle and 392.1 mm/cycle, respectively. The lowest average number of eggs was obtained in Group 1 (302.1 ± 68.62/trap; cycle 1) when the accumulated rainfall was just 18.9 mm. A low positive correlation (N = 196, r = 0.2519, p < 0.05) was observed between the number of eggs and females collected in the sites (Figure 4).

#### Other mosquito species

During the above experiments a total of 270 specimens from at least three mosquito species were captured by the AedesTrap. Of these, 47.0% were *A. aegypti*, 39.3% were *Culex quinquefasciatus*, 4.1% were *A. albopictus* and 9.6% were an unidentified species.

The proportion of mosquitoes captured by the AedesTrap varied significantly (F = 19.53; GL = 3,78; p < 0.001) among species. Individual comparison using the Tukey test showed that the number of captured *A. aegypti* was similar to *C. quinquefasciatus* (p = 0.688). However, both differed significantly (p < 0.0001) from *A. albopictus*.

#### Location of the AedesTrap: indoors and outdoors

A total of 99 *A. aegypti* females were collected over the period from April to July 2009, where the performance of the AedesTrap was evaluated indoors and outdoors. Capture rate was significantly higher (p < 0.01) in outdoor areas (73.7%) compared to indoor (26.3%), with a mean capture rate of 0.7 ± 0.1 and 0.3 ± 0.07/AedesTrap, respectively. Overall, a total of 99 *A. aegypti* females were collected over the period from April to July 2009. Thirty three percent of traps were positive outdoors, whereas 9.5% were positive indoors. Out of the 35 captured *C. quinquefasciatus*, 25 were registered outdoors.

The positivity index of the AedesTraps varied from 13% to 22% when installed indoors and from 32.1% to 47.8% when outdoors, whereas 95.6% to 100% of ovitraps were also positive, presenting an average of 709.6 ± 51.6 eggs/ovitrap/cycle. There was no positive correlation between the number of captured *A. aegypti* and the number of eggs at the sites (N = 105; rs = 0.0331; p = 0.738).

#### AedesTrap and mosquito capture numbers

**Figure 5:**
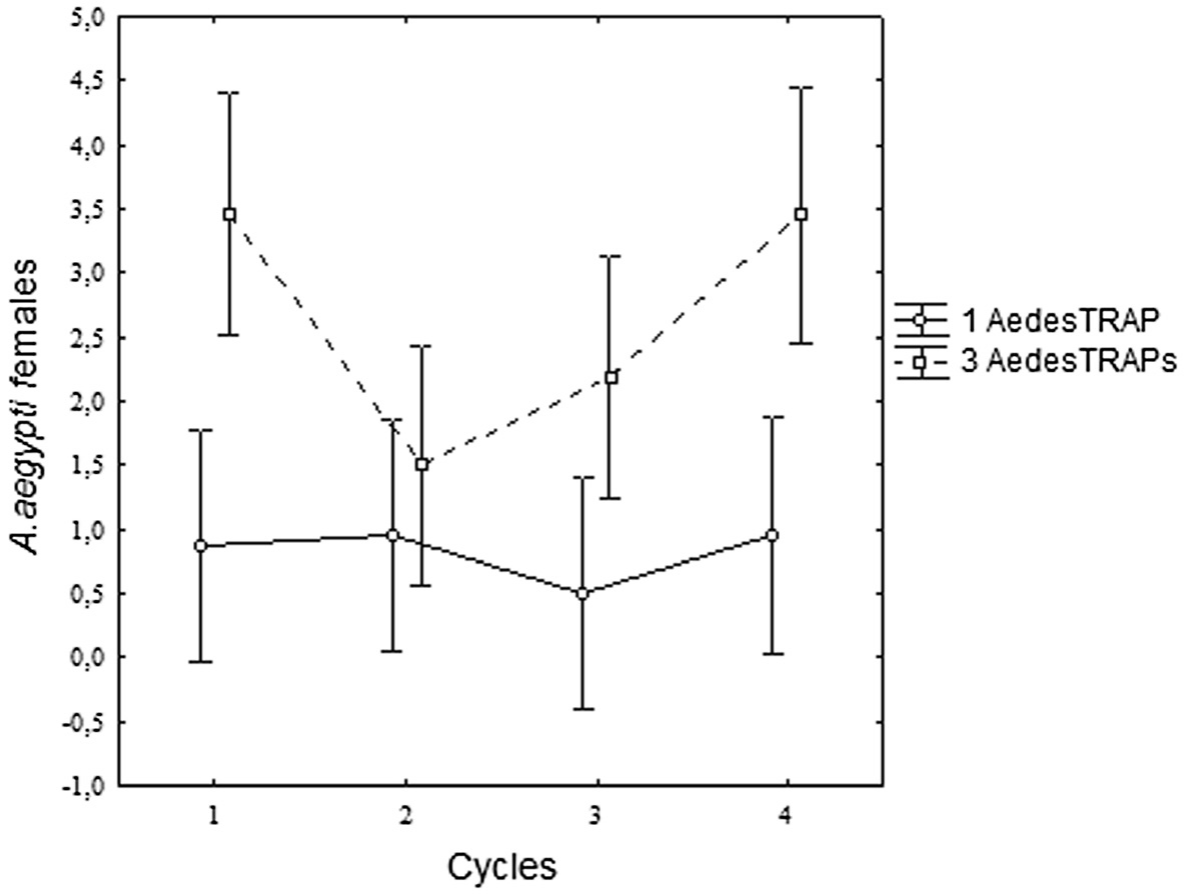
*** A. aegypti *capture using one or three AedesTrap.** Variance in capture success was calculated using the total number of *Aedes aegypti*/residence with one or three AedesTrap in the neighborhood of Engenho do Meio, Recife, Pernambuco, during four observation cycles from March to August 2009.

The presence of three sticky traps at premise significantly increased the number of *A. aegypti* females captured by the AedesTrap (F = 29.98; GL = 3,17; p < 0.01), especially during cycles 1 and 4 (Figure 5). From a total of 304 *A. aegypti* females captured during this study, 25.7% were found as sites containing one trap and 74.3% at sites with three traps. A mean of 0.82 ± 0.2 and 2.63 ± 0.27 *A. aegypti/*AedesTraps/cycle females was captured using one and three, respectively, representing a three-fold increase in the number of females removed from the environment when three traps were used. A significantly higher number (F = 12.76; GL =1.173; p < 0.01) of *C. quinquefasciatus* was also captured at sites with three AedesTraps. Table 1 shows the mean variation of mosquitoes captured in different cycles at sites having one or three AedesTraps. The installation of three AedesTraps at a site increased the possibility of discovering the presence of *A. aegypti* at the site. Using three AedesTraps/premise, 75.6% of the premises were positive, compared to 33.7% at sites with one trap. In the same period, ovitrap positivity was 99%.

**Table 1:** Relative frequency of mosquito species, mean capture rate of AedesTrap and average egg number, in premise with one or three sticky traps, between March and August 2009, in Engenho do Meio neighborhood, Recife, PE.

	**Capture and catch cycles of* Aedes *spp.**				
**Number of AedesTrap/parameters evaluated**	**Cycle 1**	**Cycle 2**	**Cycle 3**	**Cycle 4**	**Total/overall mean**
**One trap**					
n° of premises	24	23	23	22	92
n° of *A. aegypti*	21	23	12	22	78
average number of	0.88 (±0.32)	0.96 (±0.63)	0.5 (±0.25)	0.96	0.82 (±0.2)
females (±SE)/trap				(±0,27)	
average number of	0.88 (±0.32)	0.96 (±0.63)	0.5 (±0.25)	0.96	0.82 (±0.2)
females (± SE)/premise				(±0,27)	
n° of *Aedes albopictus*	0	0	0	0	0
average number of females (±SE)/trap	NE	NE	NE	NE	NE
average number of females (± SE)/premise	NE	NE	NE	NE	NE
Eggs density *Aedes* spp./premise	794.92 (±111.09)	596.29 (±96.95)	506.54 (±76.58)	602.43 (±115.29)	625.28 (± 50.77)
**Three trap**					
n° of premises	21	21	21	19	82
n° of *A. aegypti*	76	33	48	69	226
average number of females (±SE)/trap	1.12 (± 1.62)	0.5 (± 1.15)	0.73(± 1.3)	1.15 (± 1.75)	0.86 (± 1.48)
average number of females (± SE)/premise	3.45 (± 0.53)	1.50 (± 0.46)	2.18 (± 0.48)	3.45 (± 0.65)	2.63 (± 0.27)
n° of *Aedes albopictus*	0	0	1	3	4
average number of females (±SE)/trap	NE	NE	0.015( ± 0.4)	0.05( ± 0.1)	0.015 (± 0.01)
average number of females (± SE)/premise	NE	NE	0.05 (± 0.21)	0.15 (± 0.49)	0.05 (± 0.02)
Eggs density *Aedes* spp./premise (± SE)	983.77 (±237.91)	612.82 (±138.72)	550.91 (± 113.72)	484.70 (± 119.1)	662.08 (± 82.4.)

NE = not evaluated SD = standard error.

**Figure 6:**
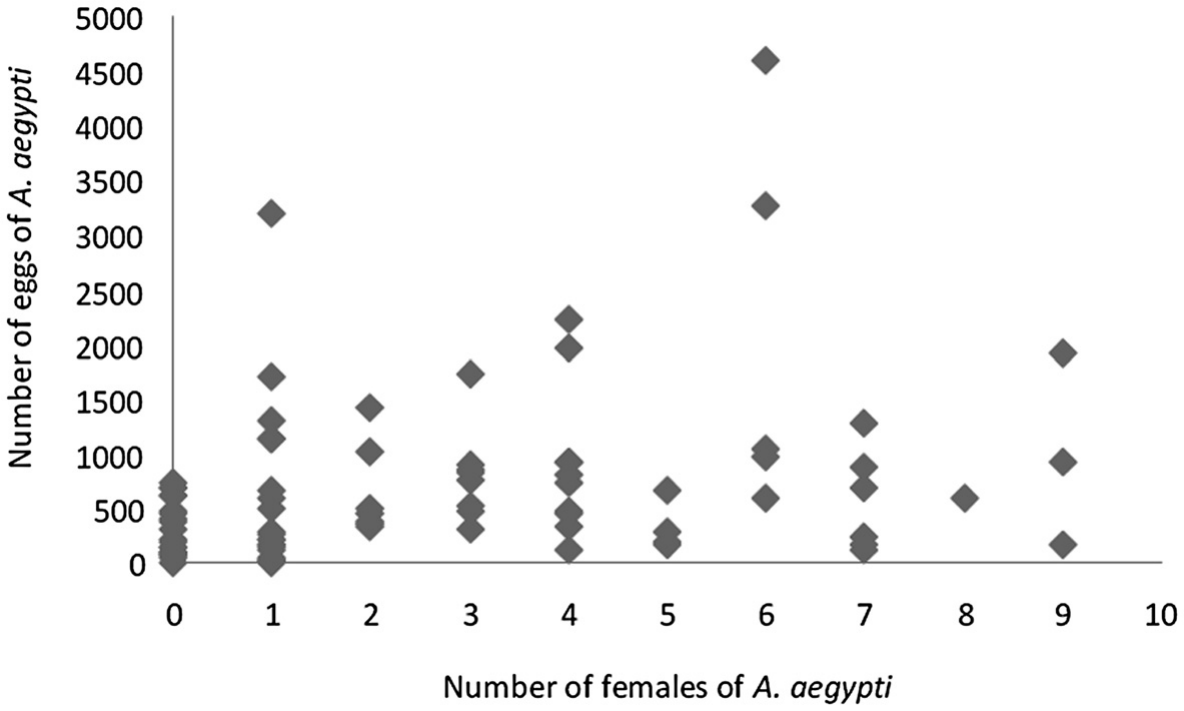
**Spearman correlation between the number of* A. aegypti *eggs and* A. aegypti *females.** Data were collected in sites with three AedesTraps/residence (N = 86; rs = 0.3920; p < 0,001) in the neighborhood of Engenho do Meio, Recife, Pernambuco.

A positive correlation (N = 86; rs = 0.39; p < 0.001) between *A. aegypti* female and egg numbers was only observed at sites with three AedesTraps (Figure 6). Egg density in ovitraps was not influenced by the number of sticky traps at the same site (Table 1). A similar mean number of eggs/ovitrap/cycle was registered at sites with one (625.28 ± 50.77) and three (662.08 ± 82.4) AedesTraps. Egg densities did not vary significantly among ovitraps installed at the sites without an AedesTrap and at sites with one AedesTrap (F = 0.00069; GL = 1,165, p = 0.9799) and three AedesTraps (F = 0.9648; GL = 1,155, p = 0.75651). During this experiment rainfall varied from 235.9 mm and 771 mm.

In all experiments, egg oviposition in water was observed by females stacked in the trap. Despite this fact no live larvae were found in these traps due to the use of *Bti* larvicide.

## Discussion

The sticky trap designed in the present work is a simple and cheap construction, which captures *A. aegypti* females. These characteristics allow its large scale usage as a complementary tool in the control of this mosquito species.

The larger number of traps in the field significantly improved AedesTrap performance in collecting *A. aegypti*. The increase in capture rates (0.8 to 2.6 mosquitoes/premise) due to the number of available AedesTraps is similar to results obtained by Wiliams et al. [21] in Australia. According to these authors, sites with 4, 6, and 8 sticky traps captured significantly more *A. aegypti* per site than sites with 1 and 2 traps. The results of the present study demonstrated that there is a strong relationship between the number of AedesTrap available and the capacity to detect mosquito infestation at sites. At all sites, ovitraps were concomitantly installed and shown to be more sensitive than AedesTraps in detecting the presence of *A. aegypti*, independent of the number of AedesTraps utilized. Studies of the performance of MosquiTrap in Belo Horizonte [10] and in Rio de Janeiro [4,14] also indicated the superior capacity of ovitrap in detecting the presence of mosquitos. According to Honório et al. [14], although both traps are sought by females as an oviposition site, differences in their attractiveness may be one of the reasons for the low efficiency of MosquiTrap.

In previous works by this group (data not shown), utilizing AedesTraps and ovitraps with hay infusion in sites with high *Aedes* spp. infestation (>3,000 eggs/premise/month), a high positivity rate (98%) was registered for both traps, differing from what was observed here, where the positivity of ovitraps was always higher than that of AedesTraps. It is worth noting that besides differences in infestation levels between sites, the continuous presence of eggs in wood paddles, as well as volatiles originating from dead larvae and bacterial fermentation of water, may have influenced the attractiveness of the ovitrap. Other authors have also referred to the stimulating characteristic of Bti for *Aedes* spp. oviposition [17,22].

Although the AedesTrap has been designed to collect adult *A. aegypti*, it also collected *C. quinquefasciatus*. The latter species is of great importance to public health in areas endemic for lymphatic filariasis, such as Recife in Brazil, as it is the vector of *Wuchereria bancrofti*, the etiologic agent of the disease [23]. The data presented here may serve as a starting point for further investigations on the use of AedesTrap to capture *C. quinquefasciatus.* This mosquito species has also been observed in other traps such as BGS-Trap [4] and Adultrap [24].

The higher positivity and capture rates of AedesTrap in outdoor areas confirmed the general consensus that this is the most appropriate location for the installation of traps, when the capture strategy is based on attracting *A. aegypti* females searching for oviposition sites [4,25,26]. In this study, about 30% of females were captured by AedesTrap exclusively outdoors relative to indoor. Similar behavior was also observed by Favaro and collaborators [27], in a study that utilized four MosquiTraps at each site and achieved 55% trap positivity per site.

Studies performed with other sticky trap models have revealed considerable variations with regard to their performance in the field, with low capture rates generally observed. Throughout the study, there was variation between traps containing *A. aegypti* and the average number of captured mosquitoes per trap. These observations were in accordance with most studies on the performance of sticky traps to monitor and control *A. aegypti*. Possible elements affecting this variance are environmental factors [24,28] and the peculiar behavioral characteristics of *A. aegypti*, such as “skip oviposition” [4,6,9,10,29]. In contrast, Chadee and Ritchie [7] described a high rate of female capture in Trinidad, West Indies, using both standard and double sticky traps. The performance of the double sticky trap was significantly superior to that of the standard model, particularly in urban areas.

The “death stress oviposition” behavior described by Chadee and Ritchie [11] when females became stuck in glue, was also observed in the present study. According to the authors, the stress caused by imminent death stimulates females to release their eggs.

The AedesTrap capacity of detecting and capturing *A. aegypti* during the evaluation of its performance was similar within different precipitation patterns, a finding that was different for ovitraps, which increased egg collection according to rainfall levels. A longitudinal study performed by Regis et al. [19], in which ovitraps were distributed in eight neighborhoods of Recife, showed a low oviposition rate in the dry season, increasing to a peak number of eggs at the beginning of the rainy period, in most areas, other than Engenho do Meio, where the present study was performed. In this neighborhood, a large number of eggs was removed from the environment with the use of more than 4,000 ovitraps per km^2^. This fact was considered as the probable reason why high egg density figures were not observed in the rainy season.

Methodological differences between this and other studies of adult sticky trap, particularly the time of traps spent in the field and the absence of attractive features in the traps used in this study, make data comparison impractical. In the present study, positivity rates and adult capture by AedesTraps used tap water, and, being monitored every 28 days did not show significant differences over the period of performance evaluation.

Studies using MosquiTrap in Belo Horizonte, in the state of Minas Gerais, showed that in the dry season (May-June), 31.5% of the traps captured 0.11 females/week [10], while in Mirasol, in the state of São Paulo, 70% of MosquiTraps (September 2006 - Mach 2008) were positive, with an average of 1.3 females/week throughout the study, with no discrimination between the mean observed in dry and rainy periods [14]. The Adultrap captured 0.18 and 1.6 females/day/trap when utilized in field tests in Foz do Iguaçu, in the state of Paraná, and in Olaria, in the state of Rio de Janeiro, respectively [9,24]. It is important to highlight that although daily or weekly observations offer more precise temporal information on infestation levels, they are logistically less viable in routine activities of entomological surveillance programs.

The use of reusable and low cost material to build traps stimulates the development of monitoring methods targeted at *A. aegypti* adults. In the present study, in which the disposable bottles were donated by restaurants, and traps were made by laboratory staff, the cost of the AedesTrap was less than U$3. This is particularly important considering the possibility of using a great number of AedesTraps in the endemic area, removing females from the environment. It is important to notice that until now there are no sensitive methods able to precisely estimate *A. aegypti* adult population density. In addition, the AedesTrap proved to be operationally viable and sustainable for monitoring *A. aegypti* adults within the conditions of health services in Brazil. Moreover, the presence of trapped mosquitoes attracted the attention of the community, a fact that could be used to enhance their effective participation in *Aedes* control programs.

## Conclusions

This study shows that the AedesTrap may be used as a tool for monitoring *A. aegypti*. The usage of at least three traps in outdoor areas allows the acquisition of more accurate data on the local adult mosquito population. This sticky trap was also capable of collecting other mosquito species such as *C. quinquefasciatus* and *A. albopictus*.

## Competing interests

The authors declare that they have no competing interests.

## Authors’ contributions

CMRA, EMMS e JCC assisted with study design, logistical issues, field data collection. All authors were involved in data analysis and writing of manuscript. The final version of manuscript was read and approved by all authors.
